# The Precautionary Principle: Radiofrequency Exposures from Mobile Telephones and Base Stations

**DOI:** 10.1289/ehp.0901370

**Published:** 2010-01

**Authors:** Stelios A. Zinelis

**Affiliations:** Hellenic Cancer Society, Cefallonia, Greece, E-mail: zinelis@otenet.gr

Dolan and Rowley (2009) reported that the precautionary principle “is not appropriate to policy on the use of mobile telephones and the siting of base stations” because there is no established health hazard from the exposure to low-dose radiation. The guidelines [International Commission on Non-Ionizing Radiation Protection (ICNIRP) 1998] provide guidance on protection only from thermal effects (when an increase in body temperature causes injury to the tissue for a short period of time). These guidelines do not cover effects on humans or the environment from nonthermal effects [i.e., effects from electromagnetic fields (EMF) or chronic exposure that do not increase body temperature]. These nonthermal effects of EMF have been well documented by Belyaev (2005) and Sage et. al. (2007). Therefore, the precautionary principle is needed to protect the environment from these effects. Several reports have recommended use of the precautionary principle for these exposures [Herberman 2008; International Commission for Electromagnetic Safety (ICEMS) 2006, 2008; Russian National Committee on Non-Ionizing Radiation Protection 2008; Sage et al. 2007]. I do not agree with Dolan and Rowley (2009) that there is no plausible hazard to humans from the exposure to low-dose radiation. Clinical diseases caused by environmental exposures develop after a long period of biochemical changes; during this time, the exposed individual may or may not have symptoms. For example, in stomach cancer, biochemical changes may occur 10–20 years before the appearance of the cancer.

Dolan and Rowley (2009) also stated that risks can be seen with other activities such as “transport (including aviation) and hot showers.” These risks result from the individual’s choices and are not comparable to exposure to electromagnetic radiation from base stations, which is a constant, chronic exposure that occurs without the individual’s knowledge and permission.

The past has taught us many lessons about risk from environmental exposures. For example, the lack of full scientific proof concerning the adverse effects of asbestos and the delay of precautionary action had devasting consequencies to human health [World Commission on the Ethics of Scientific Knowledge and Technology (COMEST) 2005]. If asbestos had been banned in 1965, when the effects of asbestos on mesothelioma were plausible but unproven, the Netherlands alone would have saved approximately 52,000 victims and €30 billion for 1969–2030. An estimated 250,000–400,000 deaths from mesothelioma, lung cancer, and asbestosis caused by past asbestos exposure will occur the next 35 years in the European Union (COMEST 2005).

In conclusion, concerning the exposure to electromagnetic fields, the precautionary principle should be applied to protect humans from environmental effects of non-thermal mechanisms.
